# Understanding the Relationship Between Mood Symptoms and Mobile App Engagement Among Patients With Breast Cancer Using Machine Learning: Case Study

**DOI:** 10.2196/30712

**Published:** 2022-06-02

**Authors:** Anna N Baglione, Lihua Cai, Aram Bahrini, Isabella Posey, Mehdi Boukhechba, Philip I Chow

**Affiliations:** 1 Department of Engineering Systems and Environment University of Virginia Charlottesville, VA United States; 2 Meinig School of Biomedical Engineering Cornell University Ithaca, NY United States; 3 Center for Behavioral Health & Technology School of Medicine University of Virginia Charlottesville, VA United States

**Keywords:** breast cancer, digital intervention, mobile intervention, mobile health, mHealth, app engagement, user engagement, mental health, depression, anxiety

## Abstract

**Background:**

Health interventions delivered via smart devices are increasingly being used to address mental health challenges associated with cancer treatment. Engagement with mobile interventions has been associated with treatment success; however, the relationship between mood and engagement among patients with cancer remains poorly understood. A reason for this is the lack of a data-driven process for analyzing mood and app engagement data for patients with cancer.

**Objective:**

This study aimed to provide a step-by-step process for using app engagement metrics to predict continuously assessed mood outcomes in patients with breast cancer.

**Methods:**

We described the steps involved in data preprocessing, feature extraction, and data modeling and prediction. We applied this process as a case study to data collected from patients with breast cancer who engaged with a mobile mental health app intervention (IntelliCare) over 7 weeks. We compared engagement patterns over time (eg, frequency and days of use) between participants with high and low anxiety and between participants with high and low depression. We then used a linear mixed model to identify significant effects and evaluate the performance of the random forest and XGBoost classifiers in predicting weekly mood from baseline affect and engagement features.

**Results:**

We observed differences in engagement patterns between the participants with high and low levels of anxiety and depression. The linear mixed model results varied by the feature set; these results revealed weak effects for several features of engagement, including duration-based metrics and frequency. The accuracy of predicting depressed mood varied according to the feature set and classifier. The feature set containing survey features and overall app engagement features achieved the best performance (accuracy: 84.6%; precision: 82.5%; recall: 64.4%; F1 score: 67.8%) when used with a random forest classifier.

**Conclusions:**

The results from the case study support the feasibility and potential of our analytic process for understanding the relationship between app engagement and mood outcomes in patients with breast cancer. The ability to leverage both self-report and engagement features to analyze and predict mood during an intervention could be used to enhance decision-making for researchers and clinicians and assist in developing more personalized interventions for patients with breast cancer.

## Introduction

### Background

In the United States, 1 in 8 women will receive a breast cancer diagnosis at some point in her lifetime [1]. Breast cancer is currently the leading cause of cancer death in women [2]. Patients with breast cancer encounter a range of psychosocial stressors that extend beyond the physical effects of anticancer treatment, including emotional distress, diminished well-being, and increased symptoms of depression and anxiety [3,4]. Untreated symptoms of depression and anxiety in women with breast cancer can lead to poor quality of life [5], increased mortality [6], and high economic costs [7].

Interventions that emphasize skill acquisition, such as cognitive behavioral therapy, have been shown to effectively reduce symptoms of depression and anxiety in patients with breast cancer [8,9]. However, numerous barriers prevent patients with cancer from receiving adequate treatment, including high financial [10] and time [11] costs, social stigma [12], and a severe shortage of trained psychotherapists, particularly in rural and underserved areas [13]. Combined, these barriers lead to almost half of breast cancer survivors reporting unmet psychosocial needs [14].

Increasingly, researchers are leveraging mobile phone apps to address mental health issues in patients with cancer. Apps are frequently cited as a way of extending cost-effective care [15,16]. In many cases, digital interventions (ie, web-based and app-delivered interventions) that mirror the content of in-person therapy perform just as well in reducing mood symptoms [17,18]. App-delivered interventions can decrease barriers associated with traditional in-person interventions as treatment is affordable, is readily available, offers efficient use of time (ie, no delays to begin treatment and self-pacing), and is no longer limited by factors such as geographic proximity to available psychotherapists. This is particularly relevant for women undergoing anticancer treatment regimens who may only have small pockets of unstructured time in a day. Numerous studies have validated the use of apps to reduce depression and anxiety symptoms [19,20], including in patients with breast cancer.

Although access to high-quality treatment is a major issue that app-delivered interventions are well poised to address, sustained engagement is a common problem [21]. Engagement is critical as it is necessary for treatment success, as studies have documented a dose-response relationship in app interventions [22,23]. A barrier to advancing knowledge of engagement in digital interventions is data density. It is common for app-delivered interventions to be deployed by a user when and where they are most convenient, potentially leading to a large data set. Fortunately, advances in machine learning have made it possible to analyze vast volumes of engagement data. However, translating these raw engagement data into clinically meaningful observations is an ongoing challenge in oncology research using mobile health (mHealth) tools [24]. Moreover, to date, no studies have presented a clear process for analyzing the relationship between engagement with mental health apps and outcomes in cancer populations using machine learning.

### Objectives

This study aimed to develop a process for investigating the dynamic relationship between engagement with a mental health app intervention and mood. The process involves several steps, including cleaning and preprocessing the raw app use data, extracting features of mood and engagement, and predicting moods from these features using machine learning algorithms. To demonstrate the application and potential usefulness of this process, we applied it to a limited number of newly diagnosed patients with breast cancer who participated in a 7-week trial that evaluated the efficacy of a suite of mental health apps [25].

## Methods

### A Process to Examine the Relationship Between App Engagement and Mood in Patients With Breast Cancer

#### Overview

**Figure 1:**
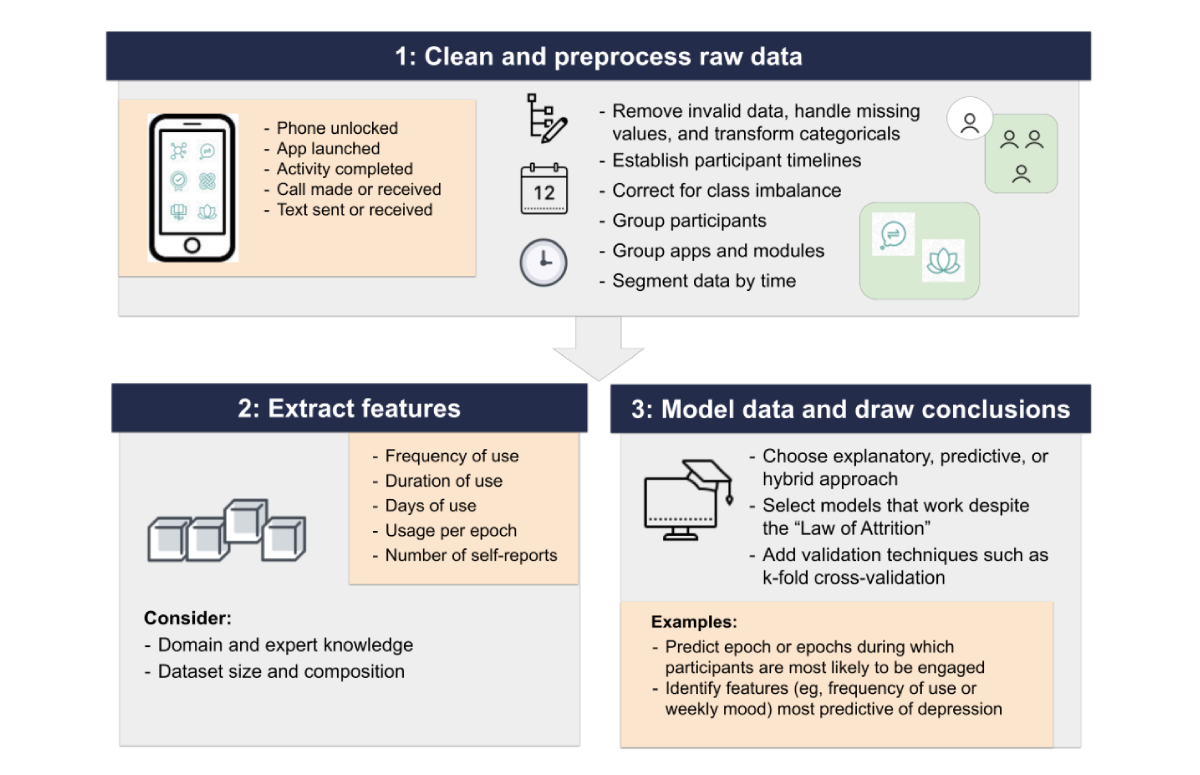
Proposed process for extracting and analyzing features of mood and engagement for patients with breast cancer using statistical and machine learning.

The overarching steps for understanding the dynamic relationship between engagement with mental health apps and mood among patients with breast cancer are outlined in Figure 1. Our process is informed by accepted data science techniques for extracting and analyzing features from raw data and gives special consideration to data sets that contain metrics of user engagement. This process assumes that researchers already have a data set that includes a mixture of time-stamped engagement data in addition to self-report data on mood. Mood data should include validated self-report measures administered at baseline, post intervention, and regular intervals (eg, weekly) throughout the study. Engagement data should comprise time-stamped event logs of app launches. It may also include information such as logs of phone lock or unlock events, mobile app launches, completed in-app activities, and outgoing or incoming calls and texts.

#### Step 1: Preprocess the Raw App Engagement Data

##### Overview

The first step is to preprocess the raw engagement data. Preprocessing is critical for preparing the data for analysis and includes removing invalid data, handling missing data, transforming categorical variables, normalizing all values, and correcting for class imbalance. In mHealth studies, such as those involving patients with breast cancer, preprocessing entails several additional tasks: establishing participant timelines, identifying time windows of interest, grouping participants, and grouping apps and modules.

##### Remove Invalid Data, Handle Missing Values, and Transform Categoricals

Invalid and missing data are common to all data sets and can occur because of user error, sensor malfunction, or lack of user action. This may be particularly relevant in the context of patients with breast cancer, given the demands and cognitive effects of treatment (eg, chemotherapy); for example, a GPS sensor may provide an inaccurate reading, or a user may complete a self-report measure on their phone but fail to click the *submit* button. Large swaths of invalid or missing data can degrade the quality of the data set and lead to less accurate analysis, making it imperative that researchers handle both with care. In mHealth studies, *invalid* data are best described as data that fall outside the acceptable range for a given variable. An example is app launches that are too short (eg, <5 seconds) or too long (eg, >5 hours) in duration. In the former case, the user opens the app and immediately closes it. In the latter case, the mobile phone sensor that monitors app use may fail to record the end of the user’s use activity period for the given app. Invalid data should be removed at the very beginning of the preprocessing stage to reduce the complexity of the data set and the computing power needed to analyze it.

*Missing values* are data that should have been recorded but were not. Newly diagnosed patients with breast cancer often struggle with both constraints on their time and the emotional burden of managing their disease [26,27]. As a result, missing data may occur at various points in a trial, such as failure to complete all administered self-report measures. Various techniques are available to account for missing data. For variables that follow a linear pattern, interpolation can be used to impute missing values between 2 time points; that is, y_i_ = (y_i-1_ + y_i+1_)/2, where the value is missing at position *i*. Alternatively, for variables with unknown or nonlinear patterns of change, more sophisticated methods such as multiple imputations using linear regression can be used [28].

After invalid and missing data are handled, categorical values from validated instruments and other self-reports should be transformed to their numeric equivalents. Finally, all data should be scaled. As these steps are not unique to mHealth or app engagement data sets, we refer to studies by García et al [29,30] for further reading.

##### Establish Participants’ Timelines

Next, individual time-stamped data points must be aligned to a standardized study timeline. Researchers often face challenges in recruiting patients with breast cancer to enroll in trials of digital interventions [31] and thus rely on a rolling enrollment period to increase recruitment over time. As a result, mHealth data sets collected from patients with cancer often have different coverage periods for each patient. Therefore, researchers must convert raw time-stamps to relative time points with respect to the study length and when a participant began the study to establish a standardized timeline for analysis. Consider 2 participants, participant A and participant B. Participant A begins the study on January 1, 2021, and submits a self-report via a mobile app on January 2, 2021. Participant B begins the study later, on January 15, 2021, and submits a self-report on January 20, 2021. Despite their different start and submission dates, both participants were said to have submitted their data during the first week of the study. This is just one example of how time-stamps may be aligned, as researchers may wish to use a different temporal granularity (eg, the day of study).

##### Consider the Issue of Class Imbalance

For studies involving classification analyses, researchers should address the issue of *class imbalance* in the data set. Class imbalance arises when observations in a small subset of categories dominate the rest [32]. This imbalance can cause problems during the analysis phase of a study by producing classifiers that always predict the dominant class or classes. Consider a study of patients with breast cancer and a simplified binary classification problem. We want to predict whether a participant is depressed given the time and frequency of app use. If most patients are depressed at baseline, the data set is *imbalanced*, and we have an overrepresentation of users with depression. As a result, a machine learning classifier may incorrectly predict that all users are depressed, irrespective of the given data. To handle this class imbalance, researchers can take what Rout et al [33] described as a *data-level approach* and either exclude some of the data of the users with depression or draw from the nondepressed users’ data to create new artificial data points. Alternatively, researchers can take an *algorithm-level* approach [33] and select a classifier that will ensure that users with depression do not skew the results. For smaller data sets, we recommend using data-level approaches such as upsampling to generate additional examples of the positive class from which an algorithm can learn. As the literature on class imbalance mitigation is broad, we refer to studies by Yap et al [34] and Rout et al [33] for more targeted reading of data- and algorithm-based techniques and strategies for selecting the most appropriate approach.

##### Group Participants

Researchers should next decide whether to group participants together or analyze engagement patterns for separate user groups. Methods of grouping participants can be broadly classified as either *theory-driven* or *data-driven*. *Theory-driven* grouping relies heavily on prior literature to categorize participants based on shared characteristics, such as demographics or mental health status. Recent studies that have grouped participants by mental health symptoms (eg, high vs low anxiety and depression) or personality traits (eg, high vs low extraversion) have revealed differences in both social and engagement behaviors between groups [35,36]. Importantly, studies in patients with breast cancer indicate a significant amount of heterogeneity in distress levels and trajectories, such that some patients experience very high levels of distress and mood symptoms, whereas others experience no or relatively low levels of distress throughout treatment [37]. On the basis of this literature, researchers may wish to classify their participants based on their baseline distress and mood scores to understand how these groups engage with mental health apps based on their differences.

*Data-driven* grouping, or *clustering,* relies on the inherent properties of a data set to identify naturally occurring groups [38]. Clustering is particularly useful for explanatory analysis of medium to large–sized novel data sets when theory-driven grouping may be infeasible. Recent research has applied clustering methods to breast cancer data sets to identify topics of conversation in breast cancer support forums [39] and investigate how depression varies according to adherence to a mood-tracking app [40]. Although outside the scope of this study, researchers seeking to conduct data-driven grouping may wish to start with 1 of the 2 common clustering methods for clinical data: *k-means clustering* or *hierarchical clustering* [41].

##### Group Apps and Modules

In studies that test >1 app or investigate an app containing multiple distinct modules, researchers must decide whether to analyze engagement in aggregate across all apps or separately for each individual app. Increasingly, researchers are developing suites of related apps that target a general domain of health, such as mental health, but have distinct target goals. In the IntelliCare suite [25], for instance, the Thought Challenger app helps users address negative thoughts, whereas the Daily Feats app helps users track their accomplishments and stay motivated. Women with breast cancer may benefit from multiple apps or a suite of apps, given their unique physical, emotional, and social needs tied to their disease. Multiple apps (or modules within a single app) that independently serve these different needs may be necessary to provide adequate support during treatment.

As with grouping participants, both theory- and data-driven grouping may be useful. For instance, theory-driven grouping can group apps according to health domain (eg, mental health) or subdomain (eg, depression management) or according to a cutoff score for a metric such as use frequency (eg, *highly used apps* are a group containing all apps used ≥6 days per week). Alternatively, data-driven clustering can be used to identify and group similar apps irrespective of the domain. Research should carefully consider the app intervention in question and whether to perform separate analyses for different groupings of apps or intervention components.

##### Segment Data by Time

Finally, researchers should consider segmenting data into meaningful windows of time or *epochs* [42]. Temporal segmentation has been used to broadly detect human activity and behavioral patterns, including facial behavior, breathing state changes [43], social behavior [35,44], and sleep disruption events [45]. Previous works within mHealth, specifically, have used theory-driven temporal segmentation to examine engagement at hourly intervals, across multihour spans (eg, *morning*, spanning 6 AM to 11:59 AM), and at weekly intervals [35,36,42,46].

When segmenting data into epochs, researchers should weigh the nature of the condition being studied and, in turn, the timescale or timescales along which symptoms and behaviors are likely to vary. Women newly diagnosed with breast cancer may only have sporadic pockets of time throughout the day to engage with a mental health app because of increased time spent attending physician’s appointments and managing their illness and sequelae of related factors. In addition, because of the disruptive impact of anxiety, depression, and cancer treatment on daily rhythms [47], patients with breast cancer experiencing mental health challenges may engage with mental health apps at irregular times. Given the stressors that patients with breast cancer face, short and frequent time windows (eg, hours or days) may be most appropriate to capture fluctuations in mood or identify the times at which a participant is most receptive to an intervention.

When segmenting their data, researchers are encouraged to balance temporal granularity against data set size. Larger data sets with more frequent measurements naturally allow for more granular epochs (eg, hourly). Researchers should also take care to ensure that epochs are neither too broad nor too narrow. Epochs that are too broad will fail to capture meaningful patterns, whereas epochs that are too narrow will introduce sparsity into the data set and decrease the effectiveness of the analysis.

#### Step 2: Extract Engagement Features

After preprocessing and before conducting machine learning classification tasks, researchers must identify the most salient variables (called *features*) within the data set and, when necessary, combine measures into new variables. This process is known as *feature extraction* and should be guided by several key factors, including domain knowledge and the size and overall composition of the data set. Importantly, researchers should avoid creating large, sparse feature sets (FSs), as this can lead to overfitting during the modeling and prediction phases. Feature extraction in small-to-medium–sized data sets, such as those of mood and app engagement, can reasonably be conducted by hand with sufficient knowledge of prior literature and the domain of interest. However, researchers interested in automated methods for high-dimensional data may find tools such as autoencoders useful [48].

Traditionally, researchers have measured engagement with blunt usage metrics such as the total or mean number of app sessions over the course of an intervention or the number of users that fail to complete an intervention [21]. However, with the increasing ubiquity of sensor-equipped smart devices, researchers have been able to derive more granular features of engagement from logs of phone or app use [49]. Several important features have emerged from recent studies, including the frequency of use (eg, number of times per week), number of days of use, duration of use, whether any use occurred in a given period, and the number of self-reports submitted [42,46,50,51]. To summarize these and other *analytic indicators of engagement*, we refer to a study by Pham et al [52].

#### Step 3: Model Data and Make Predictions

After preprocessing the data and constructing an appropriate set of features, the final step is to model and make predictions using the newly generated features. Several decisions must be made in this step. First, researchers must decide whether an explanatory, predictive, or combined modeling approach is appropriate; that is, whether the goal is to simply identify relationships between measures of engagement and mental health status or to predict one measure from another. Next, researchers must select an appropriate set of models, considering factors such as the overall data set size and structure. mHealth studies are known to have high dropout rates [21], leading to small and sparse data sets. Therefore, it is essential to select modeling techniques that can handle small data sets with a high proportion of missing or imputed data with a reasonable degree of accuracy. Finally, researchers should ensure that modeling and prediction tasks include techniques such as *cross-validation* and *parameter tuning*. Cross-validation is a technique in which random subsets of data (often multiple times) are selected as training and testing sets, which are then used to evaluate the reliability of a machine learning model [53,54]. Meanwhile, parameter tuning is the process of adjusting the model parameters to achieve better model performance metrics (eg, better accuracy and precision) [55]. Both techniques are crucial for ensuring that a machine learning model is well-constructed.

### Case Study

#### Overview

To illustrate the app engagement process, data were extracted from a 7-week trial [56] of a mobile mental health app suite among women newly diagnosed with breast cancer (N=40 participants). IntelliCare is a collection of apps that use an elemental, skills-based approach to improving mental health. In-app exercises are meant to be intuitive, requiring few instructions to complete, and most of these exercises can be found on the first screen presented by the app. Participants used their own personal phones and were recruited from a breast care clinic at a US National Cancer Institute–designated clinical cancer center. A detailed description of the recruitment method, as well as the goals of the IntelliCare apps, can be found in a paper that depicts the primary outcomes of the study [56]. Participants downloaded and tried 1 to 2 apps each week. All participants received light phone coaching that focused on addressing usability issues with the apps, which included an initial 30-minute call at the beginning of the trial, followed by a 10-minute call 3 weeks into the trial. Although 58% (23/40) of participants completed the intervention in the original trial, because of technical issues exporting app use metrics from the system, detailed app engagement data were only available for 35% (14/40) of participants.

#### Ethics Approval

This study was approved by the institutional review board at the University of Virginia (UVA IRB-HSR#20403).

#### Participant Demographics

Participants had a mean age of 56.8 (SD 11.6) years; 82% (31/38) of participants who indicated their race were White, 11% (4/38) were Black, 3% (1/38) were Hispanic, 3% (1/38) were American Indian or Alaska Native, and 3% (1/38) were multiracial.

#### Measures

The Patient Health Questionnaire-4 (PHQ-4) [57] and Patient-Reported Outcomes Measurement Information System-29 (PROMIS-29) [58] were used to assess the symptoms of depression and anxiety at baseline and after the intervention. To allow for an examination of changes in mood symptoms over the course of the trial, a 2-item measure of symptoms of anxiety and depression was administered once daily during week 1 and at the beginning of weeks 2 to 6 of the trial. The daily measures from week 1 were averaged. This measure comprised questions from the PHQ-4 (“How much did you feel nervous, anxious, or on edge?” and “How much interest or pleasure did you have in doing things?”). Both items were scored on a 5-item Likert scale (1=not at all, 2=a little, 3=somewhat, 4=quite a bit, and 5=a lot or extremely).

Weekly self-reported measures of well-being were also collected. The questions covered topics such as substance use, physical pain, connectedness to others, reception and giving of social support, general activity, and management of negative feelings. Items were scored on a 5-item Likert scale that matched the scale for the PHQ-4 and PROMIS-29 Anxiety (1=not at all, 2=a little, 3=somewhat, 4=quite a bit, and 5=a lot or extremely).

App use data were collected using the IntelliCare platform. These data contained 1 time-stamped entry per participant per app launch. Each entry included information such as the name of the app used and the launch duration in milliseconds.

#### Missingness

The rate of missing data was 39.6% among all participants (including those who dropped out at any point during the study); this rate is consistent with the often-high dropout rates in mHealth studies [21]. Among patients who completed the baseline survey, the missingness rate was 10%. Only patients who completed the baseline survey and used at least one mobile app in the IntelliCare suite were included in our final analysis (14/40, 35%).

#### Data Preprocessing and Feature Extraction

We selected 2 time windows for our analysis: the entire 7-week study lifetime and 1-week intervals (eg, week 1 and week 2). Given our overarching goal of examining the interplay between mood and engagement, we selected a theory-driven approach for grouping participants based on a wealth of literature showing that patients with breast cancer vary with regard to their distress levels and trajectory over the course of treatment. Thus, we grouped participants according to their baseline depression and anxiety symptoms and weekly mood [35,36]. For symptoms of anxiety and depression, we segmented users into *high* and *low* groups according to their baseline scores. Cutoff values for determining group placement were identified using the PHQ-4 and PROMIS-29 scoring guidelines. Users who scored ≥3 on the PHQ-4 Anxiety subscale or who scored ≥60 on the PROMIS-29 Anxiety subscale were placed in the *anxious* group, whereas the rest were placed in the group with *low anxiety*. Similarly, users who scored ≥3 on the PHQ-4 Depression subscale or who scored ≥60 on the PROMIS-29 Depression subscale were placed in the group with *high depression*, whereas the rest were placed in the group with *low depression*.

Labeling of weekly mood was conducted in a manner similar to the labeling of depression and anxiety levels at baseline. Participants with scores of ≥4 for weekly anxious mood were labeled *anxious*, and participants with scores of ≤2 for weekly depressed mood were labeled *depressed*. We note that the cutoff score for depression was applied in the inverse direction because of the nature of the question, “How much interest or pleasure did you have in doing things?”; that is, replying 1=not at all or 2=a little indicates a depressed mood.

We conducted feature extraction by hand using domain knowledge and adapting approaches from related studies. Notably, we closely followed the approach of Cheung et al [46] to quantify the metrics of engagement from logs of app use data. For instance, to calculate frequency, we grouped raw app use logs by participant and period (eg, week) and calculated the number of times the app was used during that period. We extracted 3 main measures of engagement from the raw app use data: *frequency* (number of launches), *days of use*, and *duration of use*. Variants of these measures (eg, mean frequency and duration between launches) were also included in our analysis. Table 1 provides an overview of each of the 5 FSs used in the analysis.

**Table 1:** Feature sets (FSs) used in the analysis.

FS	Description	Example features
FS1	Engagement features for all apps	Frequency of use for all apps combined, days of use, duration of use, and mean duration of use
FS2	Engagement features for only the most frequently used app or apps	Frequency of use for the app “Worry Knot” and days of use for the app “Thought Challenger”
FS3	Self-report features+engagement features for all apps	PROMIS^a^ social support score, frequency of use for all apps combined, and days of use
FS4	Self-report features+engagement features for only the most-used app or apps	PROMIS social support score, duration of use for the apps “Thought Challenger” and “Worry Knot”
FS5	Self-report features+engagement features for each individual app	PROMIS physical pain score, frequency of use for the app “Worry Knot,” and days of use for the app “Daily Feats”

^a^PROMIS: Patient-Reported Outcomes Measurement Information System.

To prepare the data for both the regression and classification tasks, we conducted multiple imputations [28] to handle missing values in self-reported measures. Class imbalance in the classification tasks was handled using the Synthetic Minority Oversampling Technique (SMOTE) [59], a technique that synthesizes new samples from the minority class feature space.

#### Modeling and Prediction

##### Explanatory Analysis of Engagement Across Baseline Affect Groups

For each measure of depression and anxiety, we graphically analyzed the distributions of engagement measures at weekly intervals for both the *low* and *high* groups. Given the size of our data set, we analyzed engagement across all apps rather than by individual or groups of apps to avoid bias because of sparsity. Furthermore, the IntelliCare apps are conceptualized as belonging to the same intervention, and individual apps target related areas of mental health. Graphical analysis revealed notable differences in engagement between the groups with low and high anxiety and between the groups with low and high depression.

##### Correlation Analysis of App Engagement and Weekly Mood

To study the correlations between app engagement metrics and weekly mood, we fit linear mixed models to account for the repeated measures within each participant, using the subject as a random effect (ie, random intercepts) and different app engagement FSs as fixed effects. Specifically, we fit linear mixed-effects models with the least absolute shrinkage and selection operator with tuned penalty parameter α and weekly anxious mood as the outcome variable on 4 FSs from Table 1 and repeated this process using weekly depressed mood as the outcome variable. Self-reported features were used as control variables.

##### Predictive Modeling of Weekly Mood

We wanted to investigate whether engagement with mobile apps can be used to predict weekly anxious and depressed moods, as specified in our process. We considered the case of depressed mood and formulated a binary prediction problem as follows: given a vector of a participant’s app use activity and survey scores for a given week, we predicted whether the participant was depressed (1) or not depressed (0).

Binary prediction problems are well-handled by tree-based classifiers. These classifiers make decisions by *splitting* into one of several paths at each decision point or *node*. Thus, possible decision paths that can be taken to reach the final prediction are akin to the branches in a tree, with possible final predictions akin to the leaves. Tree-based models are known for their inherent feature selection capabilities and robustness to small sample sizes, which makes them a good fit for our analysis. We selected 2 popular tree-based classifiers, XGBoost (XGB) and random forest (RF), and ran these with leave-one-subject-out cross-validation (LOSOCV) to predict weekly anxious mood and weekly depressed mood separately on the FS3, FS5, and FS4 FSs. LOSOCV is a variant of k-fold cross-validation, a standard technique for evaluating a model’s performance, in which the entire data set is randomly split into *k* subsets. A subset was held out for testing, whereas the rest were combined to train the model, and the process was repeated for all *k* subsets. In the same vein, LOSOCV divides the data into subsets based on subjects and follows the k-fold cross-validation process.

The model hyperparameters were tuned using *gridsearch*, which attempts many combinations of different hyperparameters to find the *optimal* combination (ie, the combination that produces a model with the best performance). In our case, we paired *gridsearch* with a variant of k-fold cross-validation called *stratified group k-fold cross-validation*. This technique is similar to LOSOCV in that it prevents data leakage by ensuring that no subject from the training set also appears in the testing set. It also has the additional benefit of creating stratified splits, such that the balance of positive and negative class labels (1 and 0 seconds) is roughly the same in the training set as in the testing set. This approach, similar to the SMOTE, helps mitigate the effects of class imbalance in smaller data sets.

## Results

### Explanatory Analysis of Engagement Across Baseline Affect Groups

**Figure 2:**
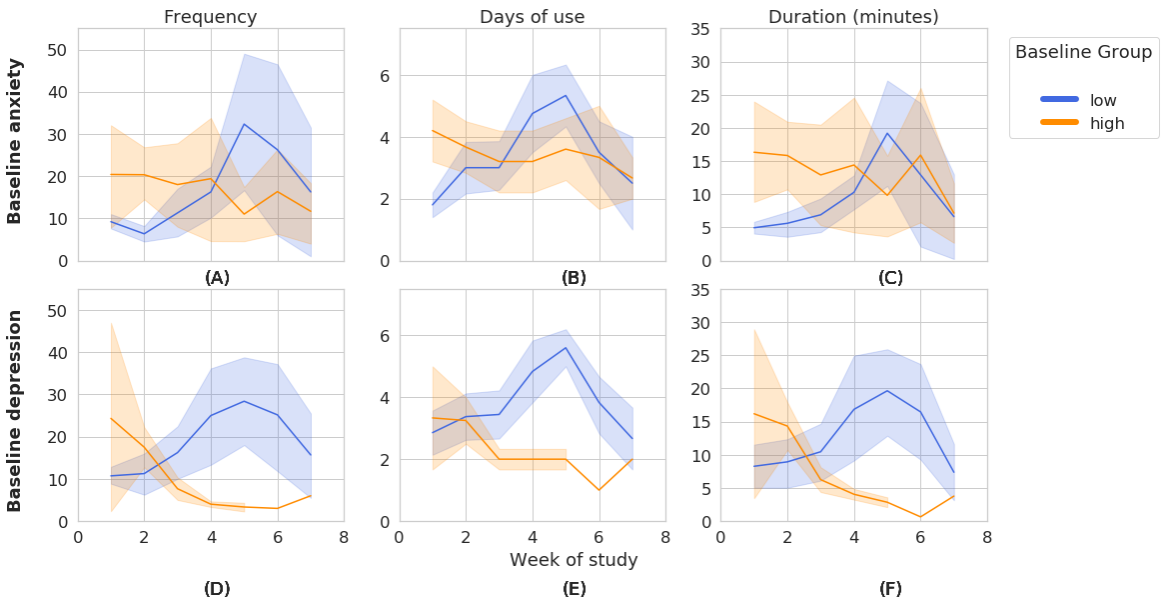
Comparison of weekly engagement metric means (with 68% CI) between 8 participants with low anxiety and 6 participants with high anxiety (A-C) and between 10 participants with low depression and 4 participants with high depression (D-F).

Both the participant groups with high anxiety and high depression experienced decreases in all 3 engagement measures between week 1 and week 7, as shown in Figure 2. Notably, the groups with high anxiety and high depression started at week 1 with higher group means than their respective *low* group counterpoints but slowly declined across measures over time. In contrast, users with low anxiety and low depression saw gradual rises across all measures, with a sharp peak around weeks 5 to 6, followed by a subsequent decrease. Interestingly, participants with low anxiety and low depression ended the study at week 7 with approximately the same group means as their respective *high* group peers.

### Correlation Analysis of App Engagement and Weekly Mood

The correlation analysis results are shown in Table 2. Several features of engagement provided significant correlations with weekly mood at *P*<.05. When engagement features for all apps were used (FS1), anxiety negatively correlated with the minimum duration (−0.0459). When features of only the most-used apps were used (FS2), depression negatively correlated with the week of study (−0.1826) and frequency (−0.1304) and positively correlated with days of use (0.4565), minimum duration (0.0414), and maximum duration (0.0248). The results for FSs FS3 and FS4 show that the inclusion of self-reported features as control variables improves model fit (indicated by root mean square error). When both self-report and engagement features for all apps were used (FS3), depression negatively correlated with frequency (−0.086), mean duration (−0.0637), and maximum duration (−0.0215) and positively correlated with total duration (0.0024), duration SD (0.098), and minimum duration (0.0978). Finally, when both self-report and engagement features for only the most-used apps were used (FS4), depression positively correlated with the minimum duration (0.0917) and maximum duration (0.0386). Interestingly, no significant correlations were observed between the selected app use features on weekly self-reported anxiety levels for FSs FS2, FS3, and FS4. We caution against overinterpreting this finding, given the limited sample size; rather, these results demonstrate the feasibility of identifying correlates with mood from heterogeneous data sets of engagement.

**Table 2:** Linear mixed model results stratified by feature set (FS) and outcome variable.

Outcome variable	FS1,^a^ coefficient (*P* value)		FS2,^b^ coefficient (*P* value)			FS3,^c^ coefficient (*P* value)			FS4,^d^ coefficient (*P* value)	
	Anxiety	Depression	Anxiety	Depression	Anxiety		Depression	Anxiety		Depression
Week of study	0 (—^e^)	−0.16 (.14)	−0.0063 (.93)	−0.1826 (<.001)^f^	0.1122 (.22)		0.0659 (.62)	0.0643 (.43)		0.1803 (—)
Frequency	−0.0169 (.55)	−0.0632 (.14)	−0.0976 (.09)	−0.1304 (.004)^f^	−0.0438 (.12)		−0.086 (.004)^f^	−0.1747 (.001)		−0.5962 (—)
Days of use	0.0761 (.53)	−0.0737 (.74)	0.1757 (.08)	0.4565 (<.001)^f^	0.1047 (.38)		0.2374 (.25)	0.2909 (.02)		1.5607 (—)
Total duration	0.0003 (.67)	0.0021 (.12)	0.0011 (.63)	−0.0017 (.17)	0.0009 (.24)		0.0024 (.01)^f^	0.0026 (.24)		0.0009 (.68)
Mean duration	0.0237 (.17)	−0.027 (.24)	0.0071 (.78)	−0.0336 (.12)	0.0007 (.97)		−0.0637 (.03)^f^	−0.0092 (.66)		−0.1536 (—)
Duration SD	−0.0172 (.36)	0.0354 (.45)	0.0055 (.83)	−0.0093 (.66)	−0.0002 (.99)		0.098 (.02)^f^	0.0026 (.91)		0.0901 (—)
Minimum duration	−0.0459 (.02)^f^	0.032 (.37)	−0.0171 (.52)	0.0414 (.03)^f^	−0.0269 (.21)		0.0978 (.01)^f^	−0.0083 (.75)		0.0917 (<.001)^f^
Maximum duration	0.0007 (.92)	−0.0105 (.44)	−0.0047 (.70)	0.0248 (<0.001)^f^	0.0004 (.95)		−0.0215 (.05)^f^	−0.0006 (.96)		0.0386 (<.001)^f^

^a^FS1: anxiety: α=.1, root mean square error 0.7396; depression: α=.1, root mean square error 0.7589.

^b^FS2: anxiety: α=.7, root mean square error 0.8095; depression: α=.1, root mean square error 1.3954.

^c^FS3: anxiety: α=.1, root mean square error 0.5128; depression: α=.1, root mean square error 0.4136.

^d^FS4: anxiety: α=.1, root mean square error 0.5348; depression: α =.1, root mean square error 0.4547.

^e^*P* value was not defined.

^f^Effects with a *P* of <.05.

### Predictive Modeling of Weekly Mood

The predictive modeling results are shown in Table 3 below. FS3, which contained survey features and overall app engagement features, achieved the highest predictive accuracy (84.6%) and yielded the best outcome measures when used with an RF classifier to predict depressed mood. FS4, which contained survey features and engagement features only from the most-used apps, achieved the second-best predictive accuracy (81.5%) when used with an XGB classifier. FS5 yielded the worst results overall, likely because of a combination of overfitting and a lack of meaningful information contained in engagement features for individual apps. Overfitting is a common issue for tree-based models applied to small data sets and occurs when the model learns the training set so well that it poorly generalizes when making predictions on the test set. We note that despite using techniques such as the SMOTE and LOSOCV, which are designed to reduce overfitting, we still struggled to mitigate this issue in our predictive task. Further investigation is warranted to determine whether a larger data set might yield better predictive results.

**Figure 3:**
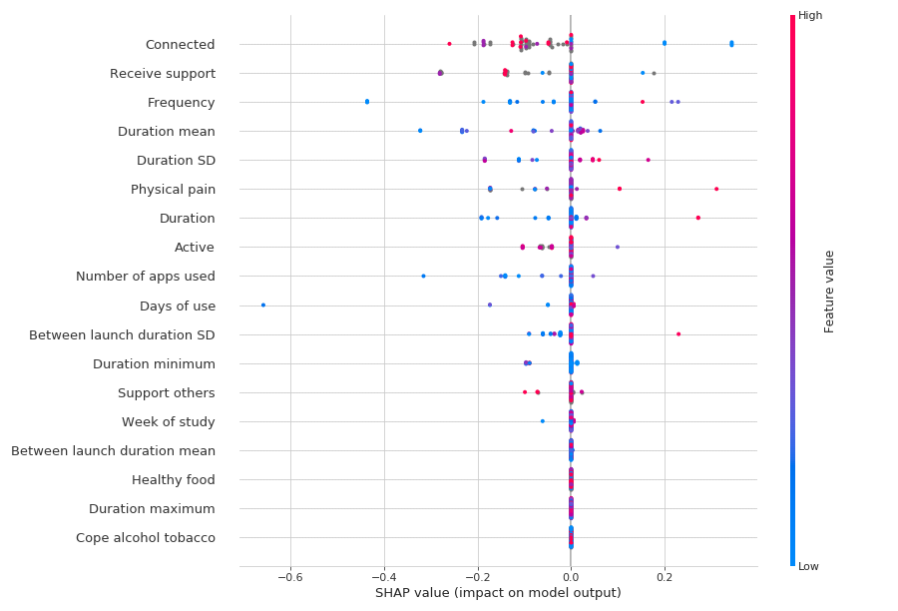
Feature importance for the prediction of depressed mood using a random forest classifier on feature set 3. SHAP: Shapley Additive Explanations.

A feature importance graph of Shapley Additive Explanations (SHAP) scores [60] for the top classifier and FS (ie, RF/FS3) for depressed mood prediction is shown in Figure 3. Self-report features such as connectedness to others (feature *Connectedness*) and receiving support from others (feature *Receive support*) were particularly important. Engagement features such as frequency and the mean duration of use were also important. As with the results of our correlation analysis, we caution against overinterpretation of the importance of individual features, given the limited sample size.

The findings from these exploratory analyses indicate that it may be feasible to identify the weekly moods of patients with breast cancer based on their app use metrics.

**Table 3:** Weekly depressed mood prediction task results.

Classifier and FS^a^		Accuracy, %	Precision, %	Recall, %	F1, %
**Random forest**					
	FS3	84.61	82.50	64.42	67.75
	FS4	83.07	73.50	72.11	72.76
	FS5	66.15	50.00	50.00	49.93
**XGBoost**					
	FS3	78.46	67.33	69.23	68.13
	FS4	81.53	70.81	62.50	64.54
	FS5	67.69	47.95	48.07	48.00

^a^FS: feature set.

## Discussion

### Principal Findings

Considering the increased sophistication of mobile devices and app-delivered interventions that can capture minute details of user engagement, there is a need to develop increasingly sophisticated frameworks to make sense of user engagement data. In this study, we proposed a process for understanding the dynamic association between app engagement and mood using machine learning. Importantly, how engagement data are processed differs from study to study. The studies by Cheung et al [46] and Pham et al [52] drew attention to these diverse data-processing approaches and the common features that characterize engagement. Our process attempts to unify the key aspects of these approaches and refocus them on data collected from patients with breast cancer. The application of the proposed process and evaluation of statistical models support the feasibility of predicting mood status based on app engagement. The analyses and results from the case study are meant to demonstrate the potential of this approach; therefore, we caution readers not to overstate the findings of our case study. Replication of the findings in a larger data set is needed to draw more firm and generalizable conclusions.

With this caveat, the application of our process to the case study data yielded some interesting preliminary findings that may be worth pursuing in future studies. The most prominent models and theories of behavioral change highlight the importance of motivational forces to sustain a behavior [61-63], such as engagement in a mental health app. Individuals with high levels of depression or anxiety symptoms are likely to experience low self-efficacy or a low perceived ability to perform a behavior, which is likely to result in poor engagement. Our results suggest that baseline levels of anxiety and depression affect patterns of engagement among patients with breast cancer, at least in the short term. The findings for the groups with high anxiety and high depression suggest that strong initial engagement does not necessarily lead to long-term engagement *growth*. In addition, the findings for the groups with low anxiety and low depression suggest that engagement may be difficult to sustain in the long term and may reach a point of diminishing returns.

The application of our process that led to the predictive results is promising in that both the RF and XGB classifiers performed well (>60% for all metrics) even with moderate amounts of data when the FS was well-curated (ie, when *FS4* and *FS3* were used). This suggests that heterogeneous FSs comprising both baseline mental health measures and engagement data may be useful for predicting weekly moods when analyzed with robust classifiers. Predictions of weekly mood can, in theory, be used to personalize interventions. A dose-response relationship has been observed in digital health interventions, making it especially important to target patients when they are most open to receiving a dose of an app-delivered intervention. Heterogeneous data sets, along with high-accuracy classifiers, could be used within a just-in-time adaptive intervention (JITAI) [64] to predict the mood of patients with breast cancer. This mood could then be cross-referenced with the patient’s schedule to identify the optimal time window for intervention delivery. Studies have also demonstrated that distress tends to spike in women around the time they receive an initial diagnosis [65,66] but that a patient’s needs change throughout the course of treatment [67-69]. Such a just-in-time adaptive intervention could be further extended to learn the mood and engagement patterns of a patient with breast cancer over time and adjust the timing of the intervention accordingly. Further research is needed to determine the feasibility of implementing such interventions in vulnerable populations.

Prior studies examining the link between engagement with mHealth tools and symptoms have historically yielded mixed results; some studies have identified a direct relationship [35,70], whereas others have identified an inverse relationship [63,71]. Although we cannot definitively quantify this relationship in our study, both our correlation and predictive analyses suggest that paring down the available features to include only the most relevant engagement data for each individual (eg, features from only the most-used apps) and combining self-report data with passively monitored engagement data may help researchers better identify significant predictors of mood.

### Limitations

There are several limitations to this study that should be considered in light of these results. The results from the case study are limited in generalizability because of the small sample size. Data sparsity was a particular challenge when we attempted to break down our time windows of interest into smaller epochs, such as 4-hour windows describing different periods of the day (eg, *morning* and late night); therefore, we had to focus on daily and weekly time windows. Similar issues with sparsity occurred when we attempted to analyze the data for each individual app in the IntelliCare suite. Furthermore, our prediction task experienced overfitting. We recommend that researchers focus particularly on recruitment and retention for similar future studies to ensure that the resultant data set is sufficiently large for granular analyses.

Our study is also limited in scope as we did not account for demographic covariates, such as age, race, or socioeconomic status, in our mixed-effects model. As demographic factors are known to play an impactful role in health outcomes, we encourage researchers to include these factors in future studies on engagement with health apps. Finally, this study focused only on patients with breast cancer; therefore, our results may not be generalizable to other patient populations with cancer or other diseases.

### Conclusions

Inspired by existing work, this study introduces a step-by-step process for investigating the relationship between mood and mobile app engagement among patients with breast cancer. We believe our process has important implications for the study of mobile app engagement among patients with breast cancer and for the study of engagement more broadly, given its flexibility and ability to handle large and dense data sets. The results from the case study suggest a need to better tailor interventions according to the baseline symptoms of depression and anxiety of patients with breast cancer. The findings from the case study also support a wider call within the field of digital interventions to advance the understanding of user engagement and attrition to sustain long-term engagement and, hence, more robust outcomes.

## Abbreviations

FS: feature set
LOSOCV: leave-one-subject-out cross-validation
mHealth: mobile health
PHQ-4: Patient Health Questionnaire-4
PROMIS-29: Patient-Reported Outcomes Measurement Information System-29
RF: random forest
SMOTE: Synthetic Minority Oversampling Technique
XGB: XGBoost

## References

[1] (2021). Breast cancer facts and statistics. Breast Cancer.

[2] (2021). Breast Cancer Statistics. Centers for Disease Control and Prevention.

[3] Weaver KE, Forsythe LP, Reeve BB, Alfano CM, Rodriguez JL, Sabatino SA, Hawkins NA, Rowland JH (2012). Mental and physical health-related quality of life among U.S. cancer survivors: population estimates from the 2010 National Health Interview Survey. Cancer Epidemiol Biomarkers Prev.

[4] Linden W, Vodermaier A, Mackenzie R, Greig D (2012). Anxiety and depression after cancer diagnosis: prevalence rates by cancer type, gender, and age. J Affect Disord.

[5] Reich M, Lesur A, Perdrizet-Chevallier C (2008). Depression, quality of life and breast cancer: a review of the literature. Breast Cancer Res Treat.

[6] Watson M, Haviland JS, Greer S, Davidson J, Bliss JM (1999). Influence of psychological response on survival in breast cancer: a population-based cohort study. Lancet.

[7] Carlson LE, Bultz BD (2004). Efficacy and medical cost offset of psychosocial interventions in cancer care: making the case for economic analyses. Psychooncology.

[8] Gudenkauf LM, Antoni MH, Stagl JM, Lechner SC, Jutagir DR, Bouchard LC, Blomberg BB, Glück S, Derhagopian RP, Giron GL, Avisar E, Torres-Salichs MA, Carver CS (2015). Brief cognitive-behavioral and relaxation training interventions for breast cancer: a randomized controlled trial. J Consult Clin Psychol.

[9] Johnson JA, Rash JA, Campbell TS, Savard J, Gehrman PR, Perlis M, Carlson LE, Garland SN (2016). A systematic review and meta-analysis of randomized controlled trials of cognitive behavior therapy for insomnia (CBT-I) in cancer survivors. Sleep Med Rev.

[10] Chi M (2019). The hidden cost of cancer: helping clients cope with financial toxicity. Clin Soc Work J.

[11] Yabroff KR, Davis WW, Lamont EB, Fahey A, Topor M, Brown ML, Warren JL (2007). Patient time costs associated with cancer care. J Natl Cancer Inst.

[12] Holland JC, Kelly BJ, Weinberger MI (2010). Why psychosocial care is difficult to integrate into routine cancer care: stigma is the elephant in the room. J Natl Compr Canc Netw.

[13] Charlton M, Schlichting J, Chioreso C, Ward M, Vikas P (2015). Challenges of rural cancer care in the United States. Oncology (Williston Park).

[14] Martínez Arroyo O, Andreu Vaíllo Y, Martínez López P, Galdón Garrido MJ (2019). Emotional distress and unmet supportive care needs in survivors of breast cancer beyond the end of primary treatment. Support Care Cancer.

[15] Muñoz RF, Chavira DA, Himle JA, Koerner K, Muroff J, Reynolds J, Rose RD, Ruzek JI, Teachman BA, Schueller SM (2018). Digital apothecaries: a vision for making health care interventions accessible worldwide. Mhealth.

[16] Firth J, Torous J, Nicholas J, Carney R, Pratap A, Rosenbaum S, Sarris J (2017). The efficacy of smartphone-based mental health interventions for depressive symptoms: a meta-analysis of randomized controlled trials. World Psychiatry.

[17] Andersson G, Cuijpers P, Carlbring P, Riper H, Hedman E (2014). Guided Internet-based vs. face-to-face cognitive behavior therapy for psychiatric and somatic disorders: a systematic review and meta-analysis. World Psychiatry.

[18] Reger MA, Gahm GA (2009). A meta-analysis of the effects of Internet- and computer-based cognitive-behavioral treatments for anxiety. J Clin Psychol.

[19] Sucala M, Cuijpers P, Muench F, Cardoș R, Soflau R, Dobrean A, Achimas-Cadariu P, David D (2017). Anxiety: there is an app for that. A systematic review of anxiety apps. Depress Anxiety.

[20] Wasil AR, Venturo-Conerly KE, Shingleton RM, Weisz JR (2019). A review of popular smartphone apps for depression and anxiety: assessing the inclusion of evidence-based content. Behav Res Ther.

[21] Eysenbach G (2005). The law of attrition. J Med Internet Res.

[22] Mattila E, Lappalainen R, Välkkynen P, Sairanen E, Lappalainen P, Karhunen L, Peuhkuri K, Korpela R, Kolehmainen M, Ermes M (2016). Usage and dose response of a mobile acceptance and commitment therapy app: secondary analysis of the intervention arm of a randomized controlled trial. JMIR Mhealth Uhealth.

[23] Zhang R, Nicholas J, Knapp AA, Graham AK, Gray E, Kwasny MJ, Reddy M, Mohr DC (2019). Clinically meaningful use of mental health apps and its effects on depression: mixed methods study. J Med Internet Res.

[24] Low CA (2020). Harnessing consumer smartphone and wearable sensors for clinical cancer research. NPJ Digit Med.

[25] Mohr DC, Tomasino KN, Lattie EG, Palac HL, Kwasny MJ, Weingardt K, Karr CJ, Kaiser SM, Rossom RC, Bardsley LR, Caccamo L, Stiles-Shields C, Schueller SM (2017). IntelliCare: an eclectic, skills-based app suite for the treatment of depression and anxiety. J Med Internet Res.

[26] Hamer J, McDonald R, Zhang L, Verma S, Leahey A, Ecclestone C, Bedard G, Pulenzas N, Bhatia A, Chow R, DeAngelis C, Ellis J, Rakovitch E, Lee J, Chow E (2017). Quality of life (QOL) and symptom burden (SB) in patients with breast cancer. Support Care Cancer.

[27] Kenne Sarenmalm E, Browall M, Gaston-Johansson F (2014). Symptom burden clusters: a challenge for targeted symptom management. A longitudinal study examining symptom burden clusters in breast cancer. J Pain Symptom Manage.

[28] van Buuren S, Groothuis-Oudshoorn K (2011). mice: multivariate imputation by chained equations in R. J Stat Soft.

[29] García S, Luengo J, Herrera F (2015). Data Preprocessing in Data Mining.

[30] García S, Ramírez-Gallego S, Luengo J, Benítez JM, Herrera F (2016). Big data preprocessing: methods and prospects. Big Data Anal.

[31] Gupta A, Ocker G, Chow PI (2020). Recruiting breast cancer patients for mHealth research: obstacles to clinic-based recruitment for a mobile phone app intervention study. Clin Trials.

[32] Sun Y, Wong AK, Kamel MS (2009). Classification of imbalanced data: a review. Int J Patt Recogn Artif Intell.

[33] Rout N, Mishra D, Mallick MK, Reddy MS, Viswanath K, KM SP (2018). Handling imbalanced data: a survey. International Proceedings on Advances in Soft Computing, Intelligent Systems and Applications: ASISA 2016.

[34] Yap BW, Rani KA, Rahman HA, Fong S, Khairudin Z, Abdullah NN (2013). An application of oversampling, undersampling, bagging and boosting in handling imbalanced datasets. Proceedings of the 1st International Conference on Advanced Data and Information Engineering.

[35] Saeb S, Lattie EG, Kording KP, Mohr DC (2017). Mobile phone detection of semantic location and its relationship to depression and anxiety. JMIR Mhealth Uhealth.

[36] Mendu S, Baglione A, Baee S, Wu C, Ng B, Shaked A, Clore G, Boukhechba M, Barnes L (2020). A framework for understanding the relationship between social media discourse and mental health. Proc ACM Hum-Comput Interact.

[37] Henselmans I, Helgeson VS, Seltman H, de Vries J, Sanderman R, Ranchor AV (2010). Identification and prediction of distress trajectories in the first year after a breast cancer diagnosis. Health Psychol.

[38] Peach RL, Yaliraki SN, Lefevre D, Barahona M (2019). Data-driven unsupervised clustering of online learner behaviour. NPJ Sci Learn.

[39] Jones J, Pradhan M, Hosseini M, Kulanthaivel A, Hosseini M (2018). Novel approach to cluster patient-generated data into actionable topics: case study of a web-based breast cancer forum. JMIR Med Inform.

[40] Kim J, Lim S, Min YH, Shin YW, Lee B, Sohn G, Jung KH, Lee JH, Son BH, Ahn SH, Shin SY, Lee JW (2016). Depression screening using daily mental-health ratings from a smartphone application for breast cancer patients. J Med Internet Res.

[41] Horne E, Tibble H, Sheikh A, Tsanas A (2020). Challenges of clustering multimodal clinical data: review of applications in asthma subtyping. JMIR Med Inform.

[42] Doryab A, Villalba DK, Chikersal P, Dutcher JM, Tumminia M, Liu X, Cohen S, Creswell K, Mankoff J, Creswell JD, Dey AK (2019). Identifying behavioral phenotypes of loneliness and social isolation with passive sensing: statistical analysis, data mining and machine learning of smartphone and Fitbit data. JMIR Mhealth Uhealth.

[43] Choudhary T, Sharma LN, Bhuyan MK, Bora K (2021). Identification of human breathing-states using cardiac-vibrational signal for m-health applications. IEEE Sensors J.

[44] Boukhechba M, Daros AR, Fua K, Chow PI, Teachman BA, Barnes LE (2018). DemonicSalmon: monitoring mental health and social interactions of college students using smartphones. Smart Health.

[45] Bublitz CF, Ribeiro-Teixeira AC, Pianoschi TA, Rochol J, Both CB (2017). Unsupervised segmentation and classification of snoring events for mobile health. Proceedings of the 2017 IEEE Global Communications Conference.

[46] Cheung K, Ling W, Karr CJ, Weingardt K, Schueller SM, Mohr DC (2018). Evaluation of a recommender app for apps for the treatment of depression and anxiety: an analysis of longitudinal user engagement. J Am Med Inform Assoc.

[47] Berger T, Hohl E, Caspar F (2009). Internet-based treatment for social phobia: a randomized controlled trial. J Clin Psychol.

[48] Meng Q, Catchpoole D, Skillicom D, Kennedy PJ (2017). Relational autoencoder for feature extraction. Proceedings of the 2017 International Joint Conference on Neural Networks.

[49] Van Gemert-Pijnen JE, Kelders SM, Bohlmeijer ET (2014). Understanding the usage of content in a mental health intervention for depression: an analysis of log data. J Med Internet Res.

[50] Greer JA, Jacobs JM, Pensak N, Nisotel LE, Fishbein JN, MacDonald JJ, Ream ME, Walsh EA, Buzaglo J, Muzikansky A, Lennes IT, Safren SA, Pirl WF, Temel JS (2020). Randomized trial of a smartphone mobile app to improve symptoms and adherence to oral therapy for cancer. J Natl Compr Canc Netw.

[51] Chien I, Enrique A, Palacios J, Regan T, Keegan D, Carter D, Tschiatschek S, Nori A, Thieme A, Richards D, Doherty G, Belgrave D (2020). A machine learning approach to understanding patterns of engagement with Internet-delivered mental health interventions. JAMA Netw Open.

[52] Pham Q, Graham G, Carrion C, Morita PP, Seto E, Stinson JN, Cafazzo JA (2019). A library of analytic indicators to evaluate effective engagement with consumer mHealth apps for chronic conditions: scoping review. JMIR Mhealth Uhealth.

[53] Maxwell AE, Warner TA, Fang F (2018). Implementation of machine-learning classification in remote sensing: an applied review. Int J Remote Sens.

[54] Arlot S, Celisse A (2010). A survey of cross-validation procedures for model selection. Statist Surv.

[55] Ramezan CA, Warner TA, Maxwell AE (2019). Evaluation of sampling and cross-validation tuning strategies for regional-scale machine learning classification. Remote Sens.

[56] Chow PI, Showalter SL, Gerber M, Kennedy EM, Brenin D, Mohr DC, Lattie EG, Gupta A, Ocker G, Cohn WF (2020). Use of mental health apps by patients with breast cancer in the United States: pilot pre-post study. JMIR Cancer.

[57] Kroenke K, Spitzer RL, Williams JB, Löwe B (2009). An ultra-brief screening scale for anxiety and depression: the PHQ-4. Psychosomatics.

[58] Craig BM, Reeve BB, Brown PM, Cella D, Hays RD, Lipscomb J, Simon Pickard A, Revicki DA (2014). US valuation of health outcomes measured using the PROMIS-29. Value Health.

[59] Chawla NV, Bowyer KW, Hall LO, Kegelmeyer WP (2002). SMOTE: synthetic minority over-sampling technique. J Artif Intell Res.

[60] Lundberg SM, Lee SI (2017). A unified approach to interpreting model predictions. Proceedings of the 31st International Conference on Neural Information Processing Systems.

[61] Bandura A (1989). Human agency in social cognitive theory. Am Psychol.

[62] Ajzen I (1991). The theory of planned behavior. Organ Behav Hum Decis Process.

[63] Champion VL, Skinner CS, Glanz K, Rimer BK, Viswanath K (2008). The health belief model. Health behavior and health education: theory, research, and practice.

[64] Nahum-Shani I, Smith SN, Spring BJ, Collins LM, Witkiewitz K, Tewari A, Murphy SA (2018). Just-in-Time Adaptive Interventions (JITAIs) in mobile health: key components and design principles for ongoing health behavior support. Ann Behav Med.

[65] Burgess C, Cornelius V, Love S, Graham J, Richards M, Ramirez A (2005). Depression and anxiety in women with early breast cancer: five year observational cohort study. BMJ.

[66] Grabsch B, Clarke DM, Love A, McKenzie DP, Snyder RD, Bloch S, Smith G, Kissane DW (2006). Psychological morbidity and quality of life in women with advanced breast cancer: a cross-sectional survey. Palliat Support Care.

[67] Vogel BA, Bengel J, Helmes AW (2008). Information and decision making: patients' needs and experiences in the course of breast cancer treatment. Patient Educ Couns.

[68] Harrison JD, Young JM, Price MA, Butow PN, Solomon MJ (2009). What are the unmet supportive care needs of people with cancer? A systematic review. Support Care Cancer.

[69] Vivar CG, McQueen A (2005). Informational and emotional needs of long-term survivors of breast cancer. J Adv Nurs.

[70] Siebenhüner AR, Mikolasek M, Witt CM, Barth J (2021). Improvements in health might contradict adherence to mobile health interventions: findings from a self-care cancer app study. J Altern Complement Med.

[71] Elhai JD, Levine JC, Dvorak RD, Hall BJ (2017). Non-social features of smartphone use are most related to depression, anxiety and problematic smartphone use. Comput Human Behav.

